# Comparative proteomic analysis provides new insights into the specialization of shoots and stolons in bermudagrass (*Cynodon dactylon* L.)

**DOI:** 10.1186/s12864-019-6077-3

**Published:** 2019-09-11

**Authors:** Bing Zhang, Jibiao Fan, Jianxiu Liu

**Affiliations:** 1 College of Animal Science and Technology, Yangzhou University, Yangzhou, 225009 China; 2 Institute of Botany, Jiangsu Province and Chinese Academy of Sciences, Nanjing, 210014 China

**Keywords:** Bermudagrass, *Cynodon dactylon*, Proteome, Stolon, Shoot, Glycolysis, Starch

## Abstract

**Background:**

Bermudagrass (*Cynodon dactylon* L.) is an important turfgrass species with two types of stems, shoots and stolons. Despite their importance in determining the morphological variance and plasticity of bermudagrass, the intrinsic differences between stolons and shoots are poorly understood.

**Results:**

In this study, we compared the proteomes of internode sections of shoots and stolons in the bermudagrass cultivar Yangjiang. The results indicated that 376 protein species were differentially accumulated in the two types of stems. Pathway enrichment analysis revealed that five and nine biochemical pathways were significantly enriched in stolons and shoots, respectively. Specifically, enzymes participating in starch synthesis all preferentially accumulated in stolons, whereas proteins involved in glycolysis and diverse transport processes showed relatively higher abundance in shoots. ADP-glucose pyrophosphorylase (AGPase) and pyruvate kinase (PK), which catalyze rate-limiting steps of starch synthesis and glycolysis, showed high expression levels and enzyme activity in stolons and shoots, respectively, in accordance with the different starch and soluble sugar contents of the two types of stems.

**Conclusions:**

Our study revealed the differences between the shoots and stolons of bermudagrass at the proteome level. The results not only expand our understanding of the specialization of stolons and shoots but also provide clues for the breeding of bermudagrass and other turfgrasses with different plant architectures.

**Supplementary material:**

**Supplementary information** accompanies this paper at 10.1186/s12864-019-6077-3.

## Background

Plant architecture is defined as the three-dimensional organization of the plant body [1]. The aboveground plant body consists of a series of similar modules known as phytomers that are produced by the shoot apical meristems and axillary meristems [2]. The phytomer consists of a leaf, a leaf attachment site including an axillary bud (node) and an associated stem segment (internode) [2, 3]. Through complex growth and development, the superposition and repetition of phytomers ultimately forms the central part of the plant body, the stems, to transport substances from leaves to roots and vice versa [3, 4]. To adapt to different living environments, many plants have evolved to form many specialized stems, including stolons, rhizomes, bulbs, tendrils and tubers [5]. However, how these specialized stems differentiate and develop remains unclear, especially at the molecular level.

As an important warm-season turfgrass species, bermudagrass (*Cynodon dactylon* L., 2n = 4x = 36) is one of the most widely used turfgrasses in home lawns, public parks, golf courses and sport fields in warm regions of the world [6]. Unlike domesticated cereal grasses such as rice, wheat and maize, bermudagrass has typical characteristics of wild grasses with both erect stems (shoots) and prostrate stems (stolons) [7]. The shoots of bermudagrass produce leaves and position them in the sunlight, whereas stolons provide bermudagrass with the ability to rapidly and colonially propagate by generating new seedlings at stolon nodes [8]. Based on the development levels of shoots and stolons, the different varieties of bermudagrass can be divided into forage-type and turf-type with different plant architectures [9, 10].

Bermudagrass is highly plastic in morphology [11]. Under shaded conditions, stolon growth is inhibited, whereas elongation of shoots and enlargement of leaves are evident [12]. Low sucrose levels can promote orthotropic (erect) growth of stolons, which behave similarly to the shoots [13, 14]. The ratio of red/far red light, can also finely regulate the differentiation of stolons and shoots through phytochrome-mediated photoassimilate partitioning [15, 16]. Phytohormones, including auxin, ethylene and gibberellin, synergistically adjust the morphology of bermudagrass by regulating the development and growth of stolons and shoots [13, 17]. These observations collectively imply that the plant architecture characteristics of bermudagrass could be modified by external factors. However, the intrinsic differences between stolons and shoots that provide the basis for morphological variation in bermudagrass are still unclear.

In recent years, high-throughput transcriptomic and proteomic analyses have provided many new insights into the growth and development of stems, especially shoots and stolons, in many plants. For example, transcriptomic analysis of in vitro cultured *Arabidopsis* stem fragments at different time points during cambium initiation resulted in the identification of both stage- and tissue-specific marker genes for different steps of the process [18]. cDNA microarray and two-dimensional gel electrophoresis (2-DE) analyses revealed 1315 transcripts and 219 proteins that were differentially expressed during the developmental transition from stolons to tubers in *Solanum tuberosum* [19, 20]. Transcriptomic analysis of stolons at three developmental stages identified 5119 differentially expressed genes and 83 differentially expressed miRNAs that are possibly involved in stolon formation in *Tulipa edulis* [21, 22]. miRNA profiling revealed that the expression levels of most miRNA molecules were higher in stolons than in shoots in *Picrorhiza kurroa* [23]. Proteomic analysis of the proximal and distal internode of stolons identified 90 proteins that may be involved in stolon development in *Fragaria ananassa* [24]. Although transcriptome analysis of two wild accessions of bermudagrass with different plant architectural characteristics revealed that light- and gravity-responsive genes were preferentially expressed in a wild bermudagrass accession with well-developed stolons and degenerate shoots [25], similar comparative studies of bermudagrass stolons and shoots in the same genetic background are still deficient.

In this study, we compared the stem internode proteomes of shoots and stolons at the same developmental stage in the bermudagrass cultivar Yangjiang for the first time. The results indicated that 376 protein species were differentially accumulated in the two types of stems. Specifically, enzymes involved in starch metabolism preferentially accumulated in stolons, whereas glycolysis-related enzymes and transport-related proteins showed relatively higher abundance in shoots. Accordingly, two enzymes, ADP-glucose pyrophosphorylase (AGPase) and pyruvate kinase (PK), which participate in starch synthesis and glycolysis, respectively, showed opposite enzyme activity ratios in the two types of stems. These results collectively suggested that carbohydrate metabolism is delicately regulated in different types of stems in bermudagrass at the proteome level.

## Results

### Morphological and anatomical differences between bermudagrass stolons and shoots

**Fig. 1:**
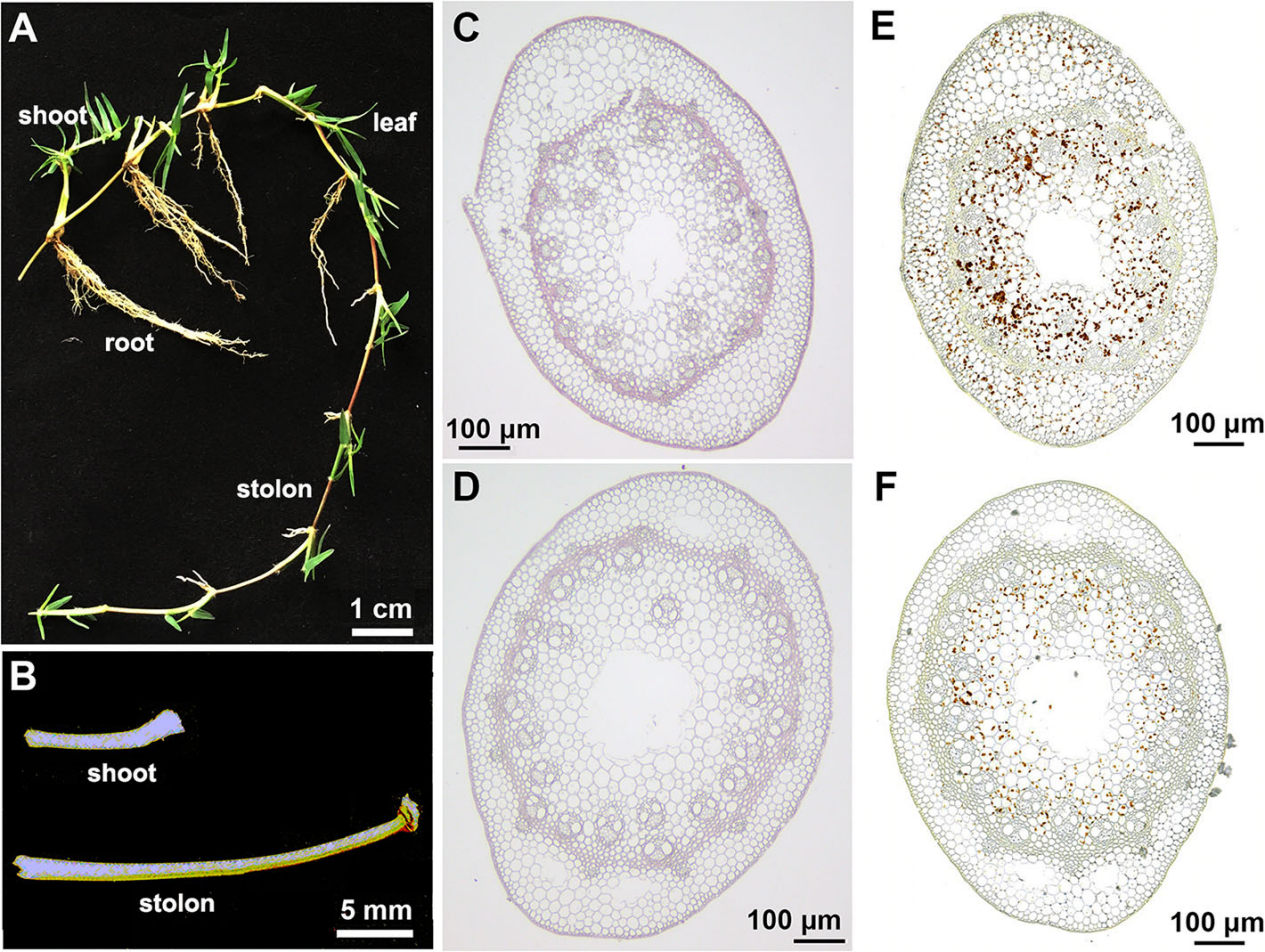
Morphology and anatomy of stolons and shoots in the bermudagrass cultivar Yangjiang. **a** Four-week-old plants and **b** the second internodes of the bermudagrass cultivar Yangjiang. Phloroglucinol staining for lignin contents in the stem internodes of **c** stolons and **d** shoots. KI-I2 staining for starch contents in the stem internodes of **e** stolons and **f** shoots. The samples shown represent five replicates per experiment

The *C. dactylon* cultivar Yangjiang is a typical turf-type bermudagrass cultivar with well-developed stolons and relatively weak shoots (Fig. 1a). Both stolons and shoots showed typical phytomer compositions including nodes and internodes. Leaves grow from the shoot nodes and new shoots sprout from stolon nodes. Interestingly, the stolons show prostrate growth, whereas the shoots show erect growth. Although the internode diameter of the two types of stems was not significantly different, the internode length of the stolons was significantly greater than that of the shoots (Fig. 1b).

To reveal the differences between the two types of stems at the cellular level, cross-sections of shoot and stolon internodes were examined under a light microscope. The volume of the shoot parenchyma cells, which are surrounded by vascular bundles, was greater than that of the stolon parenchyma cells. Lignin staining indicated that the vascular bundles and epidermis in the stolons were intensely lignified (Fig. 1c and d). Starch staining further revealed that stolon parenchyma cells accumulate more starch than those of the shoots (Fig. 1e and f).

### Stem internode proteome comparison of bermudagrass shoots and stolons

**Fig. 2:**
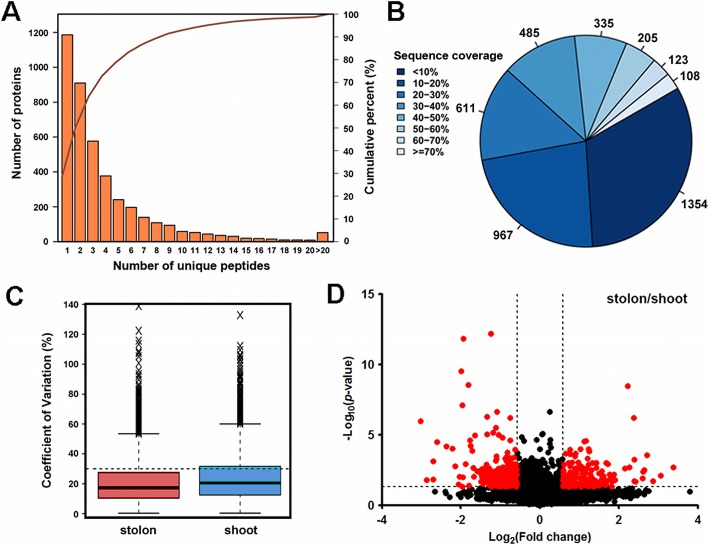
Identification of differentially accumulated protein species between shoots and stolons in the bermudagrass cultivar Yangjiang. **a** Number of protein species containing different numbers of unique peptides. **b** Number of protein species with different sequence coverages. **c** Coefficient of variation between the stolon and shoot samples. **d** Volcano plot showing the relative abundance of all identified protein species. Red and black dots represent differentially accumulated protein species and protein species with no significant difference in abundance, respectively

To explore the mechanisms underlying the morphological and anatomical differences between the shoots and stolons in bermudagrass, isobaric tags for relative and absolute quantitation (iTRAQ) proteomic analyses were performed to identify differentially accumulated proteins (DAPs) between the two types of stems. In total, 4188 protein species containing 23,581 peptides were successfully identified from 419,024 MS/MS spectra of trypsin-digested stem internode protein extracts. Among the 4188 protein species, 3002 had at least two identified unique peptides, and 1867 had a sequence coverage > 20% (Fig. 2a and b). The coefficient of variation (C.V.) was used to evaluate the reproducibility of the labeled iTRAQ samples. The results indicated that 78.02 and 68.5% of the protein species have a C.V. value < 20% in stolons and shoots, respectively (Fig. 2c). Comparisons between groups indicated that most protein species showed similar abundance in shoots and stolons, whereas only 8.98% (376) of the protein species were significantly differentially accumulated in the two types of stems (Fig. 2d). Specifically, 214 DAPs preferentially accumulated in shoot internodes, whereas another 162 DAPs showed high protein abundance in stolon internodes (Additional file 1: Table S1).

**Fig. 3:**
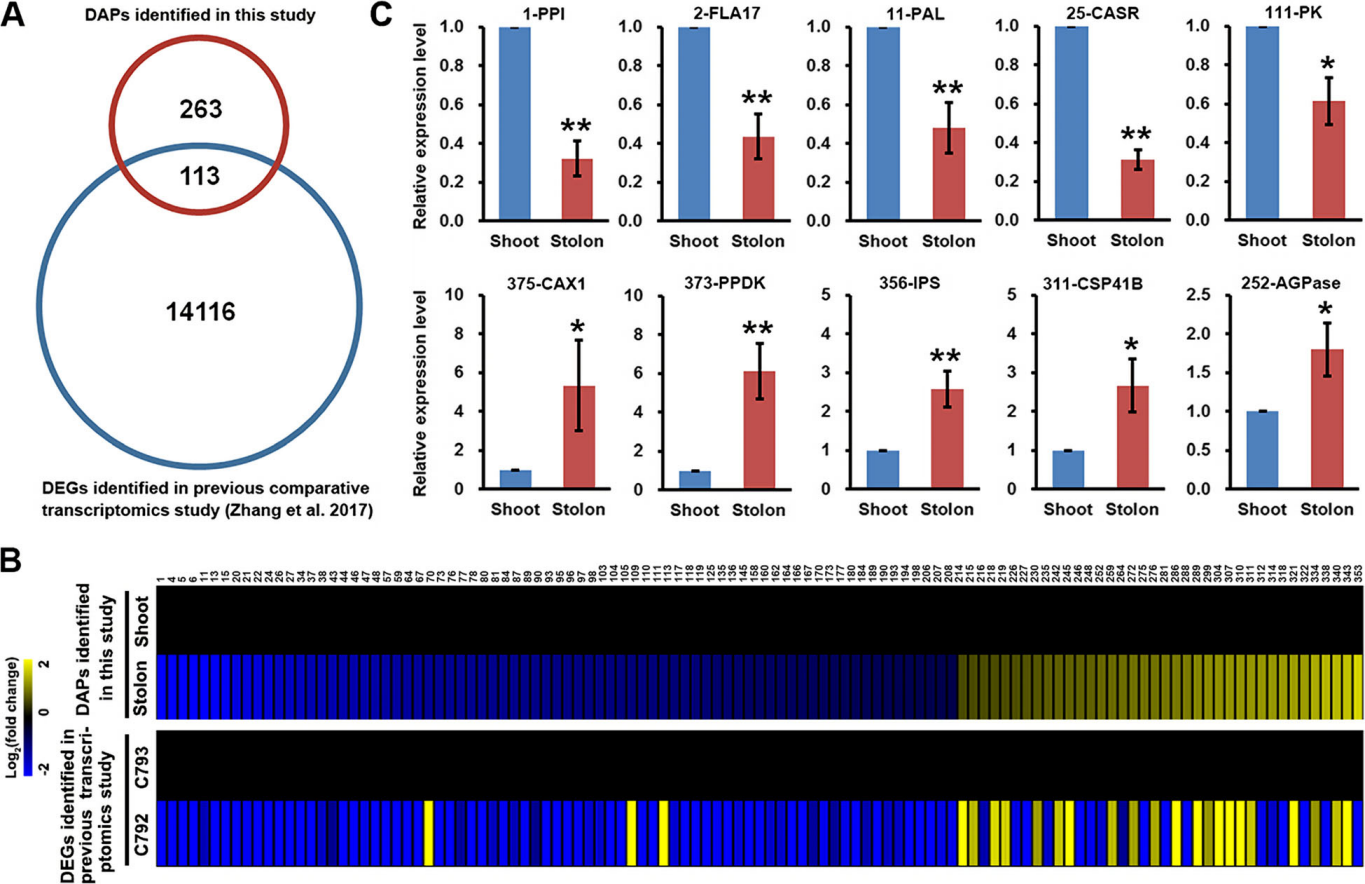
Correlation of mRNA expression levels and protein abundances of differentially accumulated protein species. **a** Venn diagram analysis of the differentially accumulated protein species identified in this study and the differentially expressed unigenes identified in a previous comparative transcriptomic study of bermudagrass stem growth directions. **b** Heat map comparison of the log_2_ transformed protein and mRNA levels between the differentially accumulated protein species identified in this study and the corresponding differentially expressed unigenes identified in the transcriptomic study. **c** RT-qPCR analysis of mRNA expression levels of ten genes encoding proteins that differentially accumulated in shoots and stolons. Error bars represent SE. Asterisks indicate significant differences determined by Student’s t-tests (*, *p* < 0.05, **, *p* < 0.01)

Interestingly, a BLAST search indicated that the genes encoding 113 DAPs were identified as differentially expressed unigenes (DEGs) in a comparative transcriptome analysis of bermudagrass wild accessions with different stem growth directions [25], whereas the genes encoding the remaining 263 DAPs were not identified as DEGs (Fig. 3a; Additional file 1: Table S1). Comparison of the iTRAQ quantification results with the transcriptome dataset indicated that as many as 93 DAPs and the corresponding DEGs exhibited similar trends of protein abundance variances and mRNA expression changes (Fig. 3b; Additional file 1: Table S1). To further validate the iTRAQ quantification results, RT-qPCR was performed to determine the expression levels of ten genes in the two types of stems. The results indicated that five genes were preferentially expressed in shoots and the other five genes were highly expressed in stolons, which was in line with the iTRAQ quantification results (Fig. 3c; Additional file 1: Table S1). However, the fold changes of these genes in the two types of stems were different for mRNA versus protein levels, suggesting that expression of these genes was post-transcriptionally regulated.

### Differentially regulated biochemical pathways in the internodes of bermudagrass shoots and stolons

**Fig. 4:**
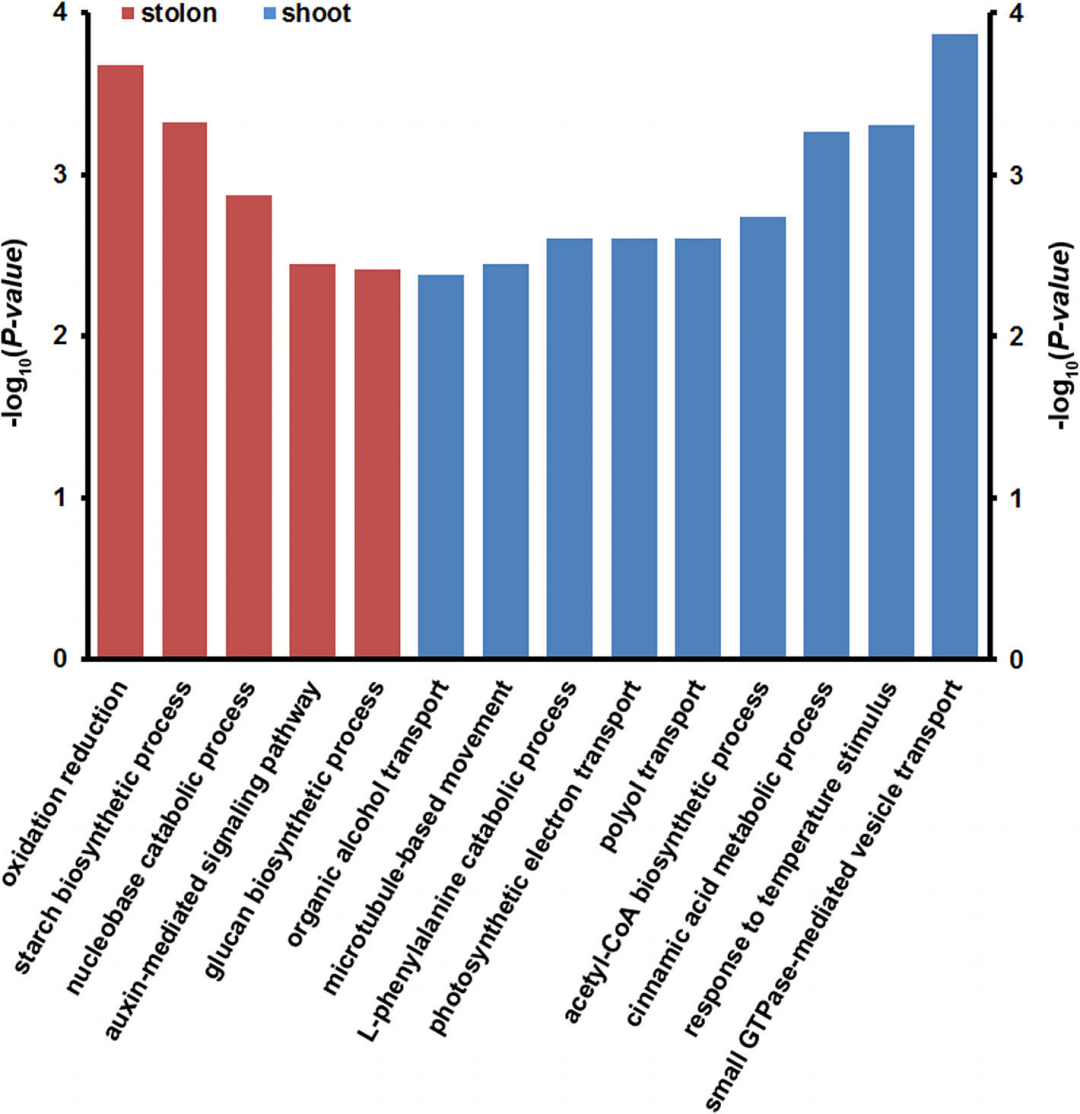
Significantly enriched biochemical pathways in the stolons and shoots of the bermudagrass cultivar Yangjiang

To determine the potential functions of the DAPs, 376 DAPs were annotated using the Gene Ontology (GO) and Kyoto Encyclopedia of Genes and Genomes (KEGG) databases. GO annotation indicated that the 376 DAPs could be classified into 29 classes (Additional file 4: Figure S1). The 29 GO classes covered many important biological and cellular processes, including metabolism, transport, growth, development, transcription, signal transduction, and response to stimulus. These results implied that many important biological and cellular processes are differentially regulated in shoots and stolons, which is in agreement with the significant morphological, anatomical and functional differences between the two types of stems (Fig. 1). KEGG Orthology Based Annotation System (KOBAS) analyses further revealed that five and nine pathways were significantly regulated in stolon and shoot internodes, respectively (Fig. 4). Specifically, oxidation reduction, starch biosynthetic process and auxin-mediated signaling pathway were significantly regulated in stolons, whereas small GTPase-mediated vesicle transport, response to temperature stimulus, polyol transport, L-phenylalanine catabolic process, and microtubule-based movement were all significantly regulated in shoots. These biochemical pathways might play important roles in the specialization of the two types of stems.

**Fig. 5:**
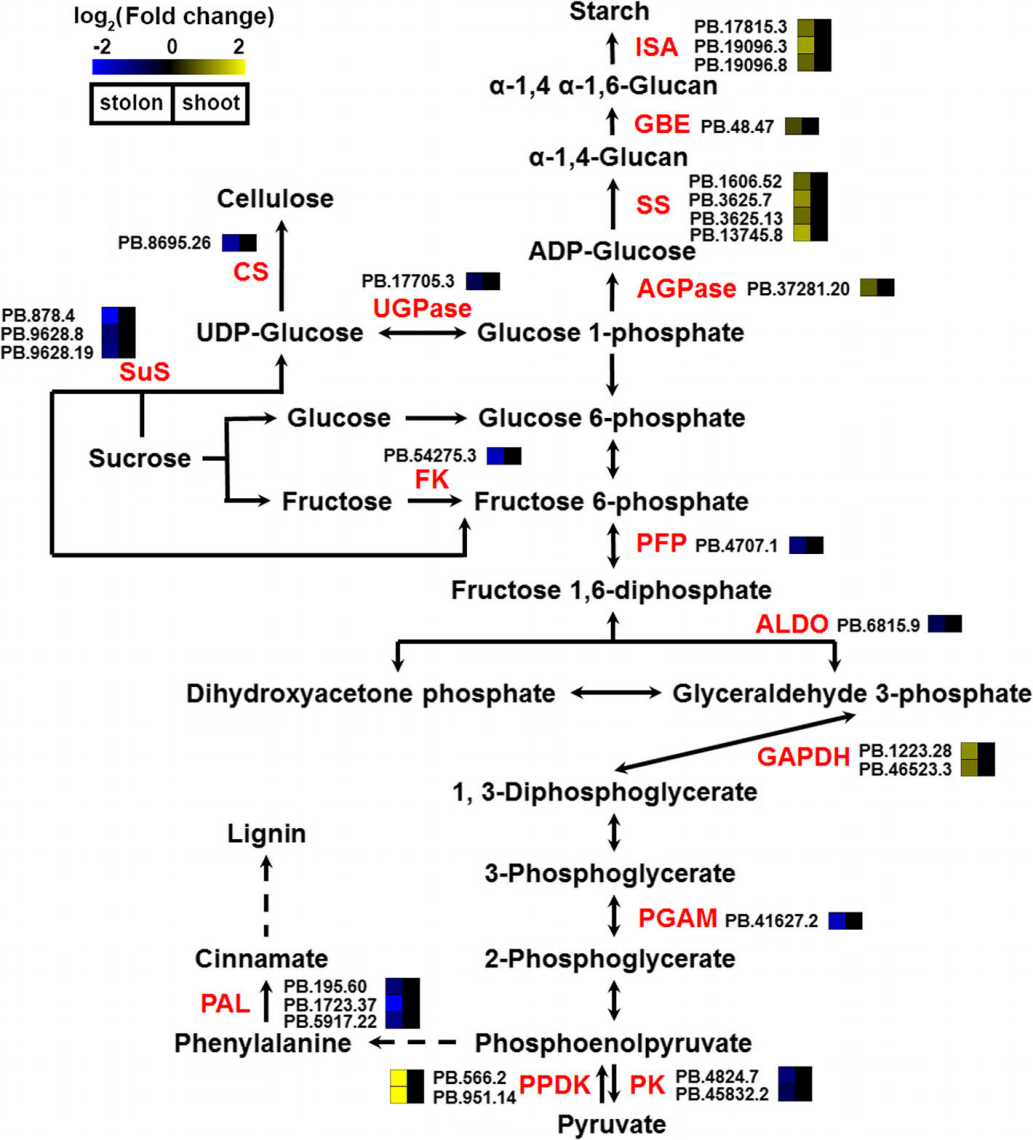
Differentially accumulated protein species involved in carbohydrate metabolism pathways. The differentially accumulated protein species identified in this study are indicated in red. Heat maps indicate the log_2_ transformed protein abundance ratio of stolons/shoots

The differential accumulation of starch was one of most prominent differences between shoots and stolons in bermudagrass (Fig. 1e and f). In accordance with these observations, enzymes that catalyze the essential steps of glycolysis, including pyrophosphate--fructose 6-phosphate 1-phosphotransferase (PFP), fructose-bisphosphate aldolase (ALDO), phosphoglycerate mutase (PGAM) and PK, all showed higher protein abundance in shoots than in stolons (Fig. 5). Enzymes that catalyze the degradation of sucrose and the synthesis of cell wall components (cellulose and lignin), including three isoforms of sucrose synthase (SuS), three isoforms of phenylalanine ammonia-lyase (PAL), a UDP-glucose pyrophosphorylase (UGPase) and a cellulose synthase (CS), were also preferentially accumulated in shoots (Fig. 5). In contrast, enzymes participating in starch synthesis, including AGPase, starch synthase (SS), 1,4-alpha-glucan branching enzyme (GBE) and isoamylase (ISA), showed higher protein abundance in stolons (Fig. 5).

### Enzyme activities of AGPase and PK in bermudagrass shoots and stolons

**Fig. 6:**
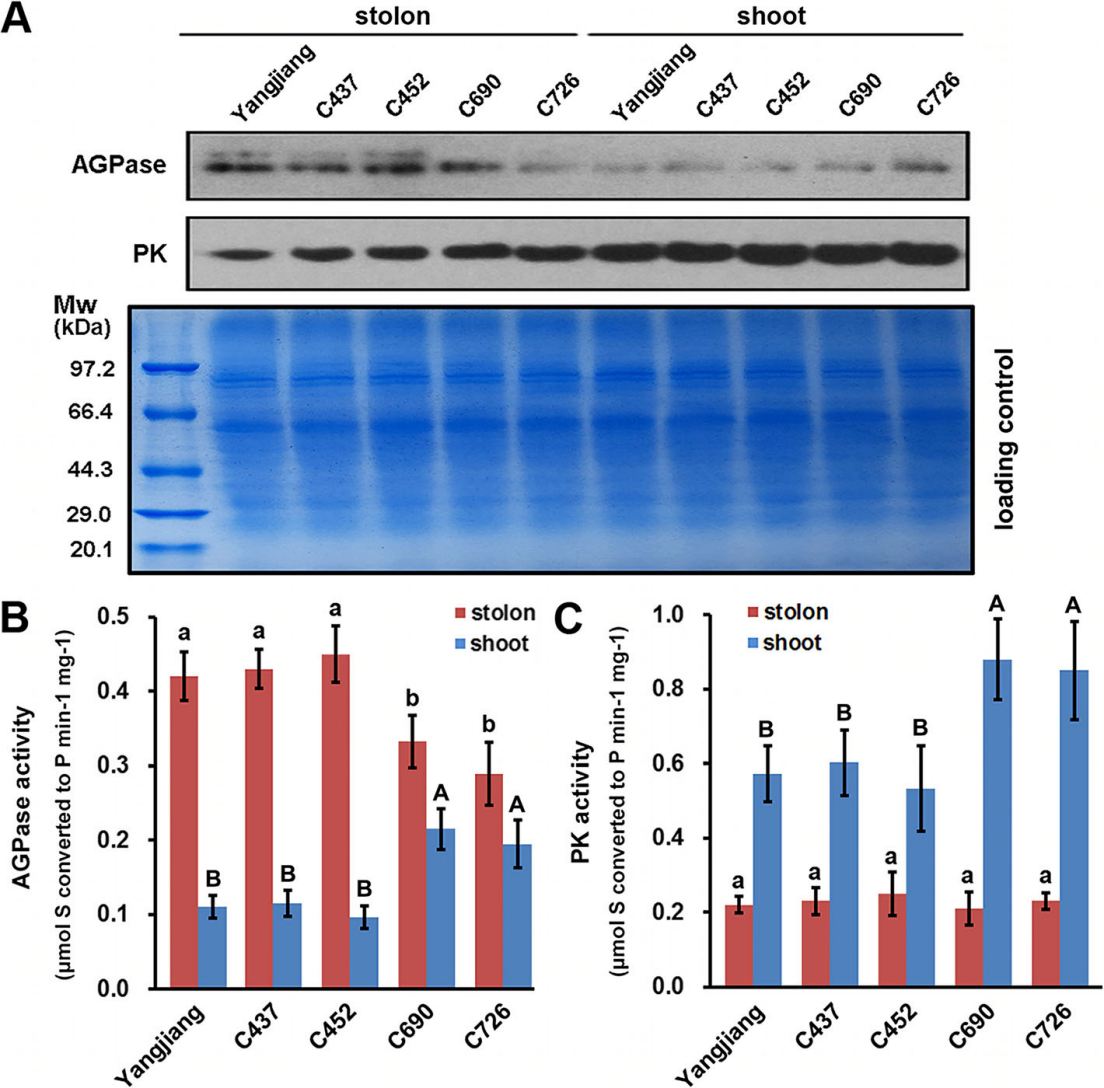
Protein abundance and enzyme activity of AGPase and PK in the bermudagrass cultivar Yangjiang and wild accessions C437, C452, C690, and C726. **a** Western blot analyses of AGPase and PK protein abundance in the stolons and shoots of five bermudagrass plants. The experiments were repeated three times with similar results. **b** AGPase and C PK enzyme activities in the stolons and shoots of five bermudagrass plants. Error bars represent SE. Values labeled with the same letter are not different at the 0.05 level of probability

AGPase and PK are two rate-limiting enzymes of the starch synthesis and glycolysis pathways, respectively [26, 27]. To further confirm the iTRAQ quantification results, the protein abundance of AGPase and PK in the bermudagrass cultivar Yangjiang was examined by western blot analysis. The results indicated that AGPase and PK were preferentially expressed in stolons and shoots, respectively (Fig. 6a). Similar results were also observed in the other four wild accessions of bermudagrass. Furthermore, the stolons of the cultivar Yangjiang and wild accessions C437 and C452 exhibited higher AGPase protein abundance than those of wild accessions C690 and C726, both of which presented relatively higher AGPase expression in shoots (Fig. 6a).

The enzyme activity of AGPase and PK in the stolons and shoots of different bermudagrass plants was also determined (Additional file 2: Table S2). In agreement with the iTRAQ quantification and western blot analysis results, AGPase activities were higher in stolons than in shoots, which exhibited higher PK activity (Fig. 6b and c). Furthermore, the difference in AGPase activities between the stolons and shoots of the cultivar Yangjiang and wild accessions C437 and C452 was greater than that between the stolons and shoots of wild accessions C690 and C726, whereas the last two wild accessions showed greater differences in PK activities between the two types of stems (Fig. 6b and c). These results, in combination with the KOBAS analysis results, collectively implied that starch synthesis and glycolysis are highly active in stolons and shoots, respectively.

### Soluble sugar and starch contents in bermudagrass stolons and shoots

**Fig. 7:**
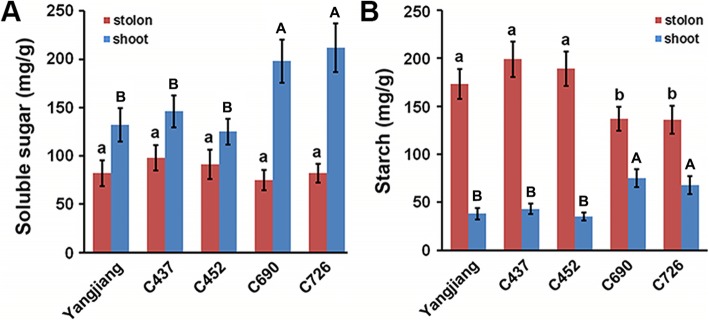
Carbohydrate contents in the bermudagrass cultivar Yangjiang and wild accessions C437, C452, C690, and C726. **a** Soluble sugar and **b** starch contents in five bermudagrass plants. Error bars represent SE. Values labeled with the same letter are not different at the 0.05 level of probability

The contents of soluble sugar and starch in the shoots and stolons of the five bermudagrass plants were further determined. In all five plants, the shoots contained more soluble sugar and less starch than the stolons, which is consistent with the starch staining results (Fig. 7a and b). Specifically, all five bermudagrass plants exhibited similar soluble sugar contents in the stolons, whereas wild accessions C690 and C726 presented significantly higher shoot soluble sugar contents than the other three plants (Fig. 7a). Similarly, the shoot starch contents of wild accessions C690 and C726 were 1.5-fold higher than those of the cultivar Yangjiang and wild accessions C437 and C452. In contrast, wild accessions C690 and C726 exhibited significantly lower stolon starch contents than the other three bermudagrass plants (Fig. 7b).

**Fig. 8:**
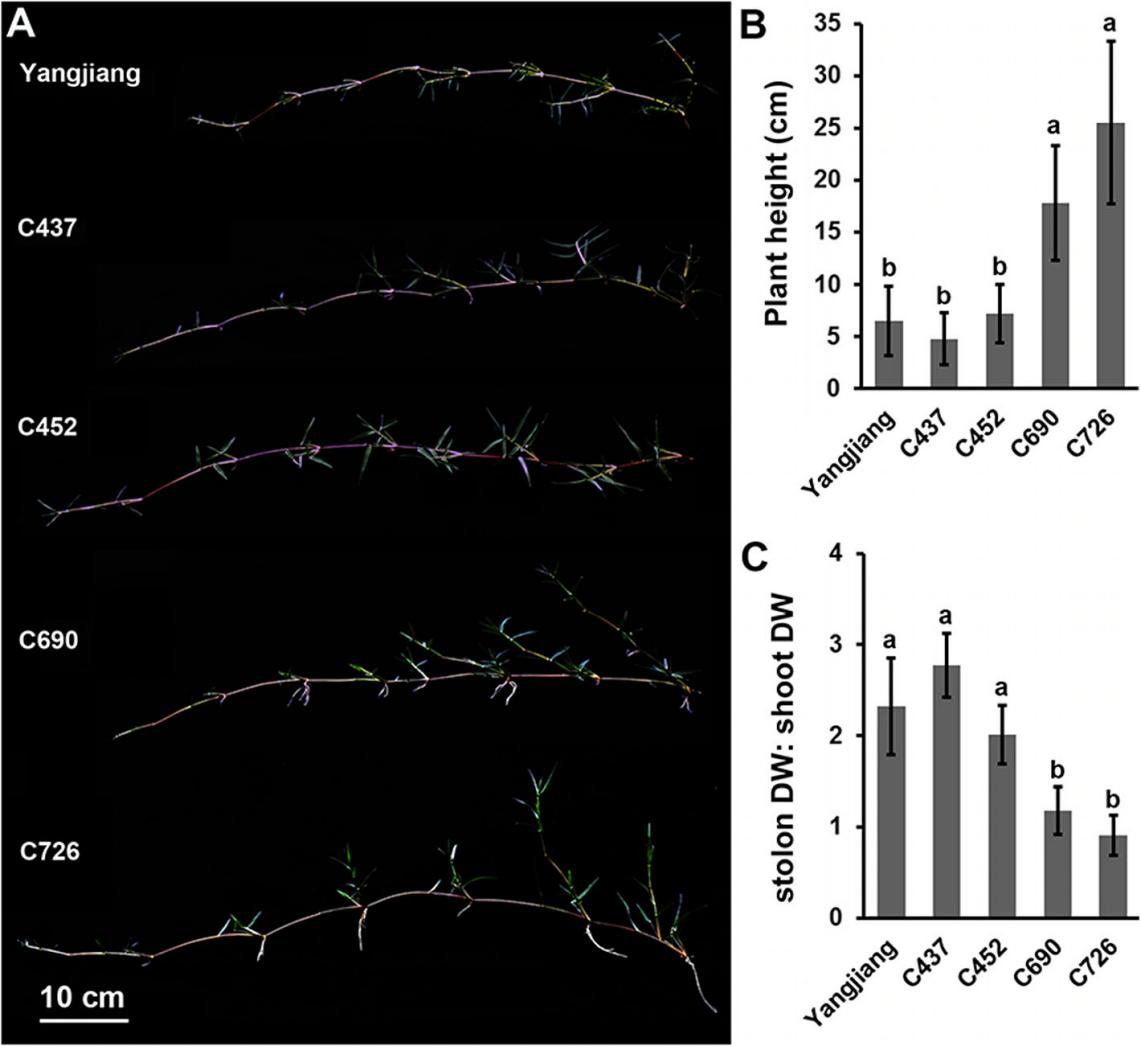
Phenotype of the bermudagrass cultivar Yangjiang and wild accessions C437, C452, C690, and C726. **a** Photographs showing the stolons of five bermudagrass plants 6 weeks after implantation. **b** Average plant height of five bermudagrass plants. **c** Comparison of stolon dry weight with shoot dry weight in five bermudagrass plants. Error bars represent SE. Values labeled with the same letter are not different at the 0.05 level of probability

Interestingly, the five bermudagrass plants also showed significant morphological variations (Fig. 8a). Specifically, shoot height was different in the five plants (Fig. 8a). The highest shoots reached 25 and 18 cm in wild accessions C690 and C726, respectively, which are both significantly higher than the heights of the cultivar Yangjiang and wild accessions C437 and C452 (Fig. 8b). Accordingly, the ratio of stolon/shoot dry weight was significantly lower in wild accessions C690 and C726 than in the other three plants (Fig. 8c). These results implied that bermudagrass varieties that accumulate and consume more sugar in their shoots grow higher than those that store more starch in the stolons.

## Discussion

Bermudagrass is a widely used turfgrass species with strong vegetative propagation ability, which depends on the continuous tillering of stolon nodes and fast growth of shoots [28]. In the past several years, high-throughput comparative proteomic analyses have been successfully performed to dissect the systematic proteome-level responses of bermudagrass during different physiological processes. For example, proteome variance under drought/salt and cold stresses was characterized in bermudagrass using 2-DE and iTRAQ, respectively [29–32]. Furthermore, the effects of exogenous molecules such as calcium chloride, polyamine and melatonin on different abiotic stress responses in bermudagrass were also analyzed at the proteome level [33–35]. In this study, we reported the comparison of the morphology, anatomy and proteome between the shoots and stolons of bermudagrass under normal growth conditions for the first time (Figs. 1 and 2). The results of this study not only expanded our understanding of the fast clonal growth of bermudagrass but also provided new insights into the specialization of stems in plants.

Comparative transcriptome analysis of two bermudagrass wild accessions with extremely different stem growth directions identified 14,229 DEGs [25]. In this study, iTRAQ analysis revealed that only 376 protein species were differentially accumulated in shoots and stolons. A BLAST search indicated that 30% of DAPs have corresponding DEGs in the transcriptome dataset, whereas up to 70% of DAPs were only identified in this study (Fig. 3a; Additional file 1: Table S1). The expression trends of 93 DAPs and the corresponding DEGs were consistent between mRNA and protein levels, whereas 20 DAPs/DEGs showed discordant fold changes (Fig. 3b). Furthermore, RT-qPCR analyses indicated that the genes encoding the ten selected DAPs also showed significant expression level changes between shoots and stolons; however, the fold changes were different between mRNA and protein levels (Fig. 3c; Additional file 1: Table S1). Similar discrepancies in transcriptome/mRNA and proteome/protein profiles have been found in *Alternanthera philoxeroides* stems under low-potassium stress and in elongating maize stem internodes [36, 37], suggesting that post-transcriptional regulation is a widespread phenomenon during stem growth and development.

Auxin is an important plant hormone that is implicated in many aspects of plant growth and development [38]. It is noteworthy that auxin-mediated signal transduction pathways were significantly regulated in bermudagrass stolons (Fig. 4). Three DAPs, including DAP no. 242 (a nucleoside diphosphate kinase 3-like protein), DAP no. 291 (a membrane steroid-binding protein 2-like), and DAP no. 356 (an inositol-3-phosphate synthase), which all participate in the regulation of auxin polar transport [39–41], were preferentially accumulated in stolons (Additional file 1: Table S1). In *Pisum sativum*, inhibition of local auxin synthesis results in a drastic reduction of the starch content of the seeds [42]. In *Phaseolus vulgaris*, the application of auxin to a decapitalized stem segment prevents the degradation of starch and promotes the lignification of the internode [43]. These results, in combination with the observation that stolons accumulated more starch than shoots (Fig. 1e and f), strongly suggested that auxin polar transport to the stolon internodes could promote starch accumulation.

As an important carbohydrate storage reserve in plants, starch is synthesized through a complex pathway regulated by multiple enzymes and primarily accumulates in many storage organs, including the tubers of potatoes, the storage roots of sweet potatoes and the bulbs of lilies [44–46]. Storage starch is stored over the dormant seasons to fuel regrowth or seedling establishment at the start of the growing season [47]. In this study, KOBAS analyses indicated that the starch biosynthetic process was significantly regulated in the stolons of bermudagrass (Fig. 4). Interestingly, enzymes catalyzing starch synthesis from photoassimilate were all highly accumulated in stolons, whereas the enzymes involved in glycolysis that consume photoassimilate to generate energy and synthesis of cell components required for cell growth and proliferation were all preferentially accumulated in shoots (Fig. 5). Accordingly, APG and PK, two rate-limiting enzymes of the starch synthesis and glycolysis pathways [26, 27], showed high enzyme activity in stolons and shoots, respectively (Fig. 6). These results collectively implied that photoassimilate metabolism was differentially regulated in stolons and shoots at the proteome level. The organized regulation of photoassimilate metabolism finally led to the massive accumulation of starch in the stolons (Figs. 1 and 7). Storage starch in the stolons could provide necessary carbohydrate nutrition to the new tillers growing out from the stolon nodes, which is important for the fast clonal growth of bermudagrass. On the other hand, high glycolysis activity could promote the fast growth of shoots and leaves by providing energy and metabolic intermediates [48].

KOBAS analyses also revealed that many intracellular and cell-to-cell transport processes including vesicle transport, polyol transport and microtubule-based movement were significantly regulated in the shoots (Fig. 4). Interestingly, seven DAPs, including DAP no. 40 (the ras-related protein RABE1c), DAP no. 77 (the GTP-binding protein SAR1A), DAP no. 133 (the ras-related protein RIC1), DAP no. 168 (the ras-related protein RABC1 isoform X1), DAP no. 171 (the GTP-binding protein SAR1A-like), DAP no. 178 (the ras-related protein RGP2), and DAP no. 204 (the ras-related protein RABH1b), which are all involved in small GTPase-mediated vesicle transport [49], showed higher protein abundance in shoots than in stolons (Additional file 1: Table S1). Additionally, DAP no. 46 (a voltage-gated potassium channel subunit beta protein), DAP no. 53 (an aquaporin PIP2–4 protein), DAP no. 64 (a plasmodesmata callose-binding protein 2-like), DAP no. 70 (an aquaporin PIP2–1 protein), DAP no. 88 (a polyol transporter 5 protein), and DAP no. 126 (a plasma membrane-associated cation-binding protein), which function as transporters and channels between adjacent cells [50–53], were all preferentially expressed in shoots (Additional file 1: Table S1). These findings were in accordance with the physiological functions of shoots. Bermudagrass is a C_4_ plant with high photosynthetic efficiency and a fast growth rate [54]. The growing shoots as well as the fast-growing leaves that sprout from the shoot nodes both need the shoots to transport large amounts of water and minerals from the roots [55]. Furthermore, the newly synthesized proteins, cell wall components, and membranes should also be transported to rapidly expanding and proliferating cells [56]. These processes all require shoots to maintain highly efficient intracellular and cell-to-cell transport systems.

A few DAPs that were not assigned in the 14 biochemical pathways might also play important roles in the specialization of the two types of stems. For example, DAP no. 61, the ultraviolet-B (UV-B) receptor UVR8 that participated in the phototropic response of shoots towards UV light [57], was found to preferentially accumulate in the shoots of bermudagrass (Additional file 1: Table S1). DAP no. 113, a phospholipase A1 protein which showed relatively higher protein abundance in the shoots, was involved in shoot gravitropism [58]. DAP no. 113, a WVD2-like 4 protein, was preferentially expressed in shoots to regulate their helical growth [59]. In contrast, DAP no. 298, a translationally-controlled tumor protein-like protein, which could promote cell proliferation and enhance growth [60], and DAP no. 314, an armadillo repeat-containing protein, which might promote the axillary organ development [61], both showed relatively higher protein abundance in the stolons (Additional file 1: Table S1). Further functional characterization of these proteins could help us to better understand their precise roles.

Bermudagrass is a turfgrass species that is widely distributed in the warm regions of the world [62]. Wild accessions of bermudagrass collected at different geographical sites often show enormous morphological diversity to adapt diverse environments [63–65]. Different molecular markers, including ISSR, SSR, AFLP and RAPD, have been successfully used to dissect the genetic diversity of different bermudagrass germplasm collections [66–70]. The results have collectively revealed a high level of sequence polymorphism in different bermudagrass cultivars and wild accessions. In this study, we also found that different bermudagrass plants showed significant morphological and physiological variance, including plant height, shoot/stolon ratio and carbohydrate contents (Figs. 7 and 8). Notably, we also observed that AGPase and PK, two key enzymes involved in carbohydrate metabolism, presented significantly different protein abundances and enzyme activities in different bermudagrass plants (Fig. 6). Interestingly, the protein abundances and enzyme activities of AGPase were significantly higher in the stolons of short bermudagrass plants living in low latitudes. In contrast, the expression level and activity of the PK protein were higher in the shoots of tall bermudagrass plants from high latitudes (Additional file 2: Table S2). Similar differential expression and activities of AGPase have been found in different wheat cultivars with different grain starch contents and near-isogenic tomato lines with different fruit sizes [71, 72]. Moreover, differences in PK expression and activities have been found between oil palm and date palm varieties that differ in carbon partitioning [73]. In combination with these findings, the results of our study implied that different germplasms of plants could finely regulate metabolic pathways to adapt to diverse living environments by modulating the expression and activity of rate-limiting enzymes.

## Conclusions

In summary, we successfully identified 162 and 214 protein species that preferentially accumulated in bermudagrass stolons and shoots, respectively. These protein species were enriched in five and nine biochemical pathways, respectively. Notably, enzymes involved in starch synthesis and glycolysis showed relatively higher abundances and activities in stolons and shoots, respectively. These results, in combination with the observation that stolons accumulate starch, and shoots exhibit higher soluble sugar content, strongly suggested that stolons and shoots are specialized for different physiological functions during fast colonial growth of bermudagrass.

## Methods

### Plant materials and growth conditions

The *C. dactylon* cultivar Yangjiang and the wild accessions C437, C452, C690, and C726 were used in this study (Additional file 2: Table S2). Before the experiments were conducted, the bermudagrass plants were grown in turfgrass plots of Nanjing Botanical Garden (32°02′N, 118°28′E; 30 m a.s.l.) under normal management conditions (irrigation: as required to keep the soil moist; fertilization: four times/year with compound fertilizer at a concentration of 5 g/m^2^; mowing: two times/month) for more than 10 years.

### Plant phenotype analyses

Healthy stolon nodes were cut from the original bermudagrass plants and grown in pots filled with soil (diameter 45 cm and height 60 cm) at a density of 20 nodes/pot. For each cultivar and wild accession, five pots were planted to represent five replicates. The pots were arranged in a randomized complete block design to minimize environmental influences and grown in a greenhouse that was maintained at 28–32 °C in the daytime and at 24–28 °C in the nighttime with 90% natural sunlight and a relative humidity range of 50–70%. At 6 weeks after planting, plant height was measured using a ruler and different plant tissues were collected. The length and diameter of the internodes of shoots and stolons were measured using a vernier caliper. The dry weight of each tissue was measured using an electronic balance. The ratio of stolon: shoot was calculated based on the dry weight of the two types of stems. Tukey’s multiple comparison test was used to determine significant differences.

### Microscopic analyses

The middle portions of the second internodes of the shoots and stolons from the bermudagrass cultivar Yangjiang were harvested and immersed in FAA fixation buffer for 24 h. After dehydration in an ethanol series (60, 70, 85 and 95%), the internodes were embedded in paraffin. Tissue sections (15 μm thick) were cut with a Leica VT 1000S vibrotome (Leica, Nussloch, Germany) and mounted on glass slides. The slides were stained with 0.1% phloroglucinol and KI-I_2_ solution (1% KI and 0.3% I_2_) for lignin and starch, respectively [25, 74]. The sections were observed and photographed using an Olympus BX51T microscope (Olympus, Tokyo, Japan).

### Soluble sugar and starch content determination

Soluble sugar and starch contents were determined as previously described [75]. Briefly, 0.1 g of dried shoot/stolon sample was ground to a fine powder. After washing with 100% acetone to remove the interfering pigments, the powder was dissolved in 5 ml of 80% ethanol, followed by incubation at 80 °C in a water bath for 30 min and centrifugation at 3, 000 g for 10 min. For the soluble sugar content assay, the supernatants collected by centrifugation were mixed with a five-fold volume of 1% (m/v) anthrone dissolved in H_2_SO_4_. The mixture was held in a 100 °C water bath for 10 min. The absorbance at 625 nm was determined using an Ultrospec 3300 Pro spectrophotometer (Amersham Biosciences, Uppsala, Sweden). The sugar content was calculated using the standard curve method. For the starch content assay, 3 ml of water was first added to redissolve the centrifuged pellet in a 100 °C water bath for 10 min and then 2 ml of 1.1% (v/v) HCl was added to promote the degradation of starch to soluble sugar. After centrifugation at 3, 000 g for 10 min, the same procedures were performed to determine the sugar content of the supernatants, which represents the starch content. Tukey’s multiple comparison test was used to determine significant differences.

### Protein extraction and quantification

The middle portions of the second internodes of the shoots and stolons were randomly collected from different plants of the bermudagrass cultivar Yangjiang and frozen in liquid nitrogen, then stored at 80 °C for protein extraction. Proteins were extracted using the previously described method with minor modifications [76]. Briefly, approximately 0.5 g of sample was ground in liquid nitrogen to a fine powder, and the powder was completely suspended in 10 ml of lysis buffer (7 M urea, 2 M thiourea, 4% SDS, 40 mM Tris-HCl at pH 8.5, 10 mM DTT, 2 mM EDTA and 1 mM PMSF) with sonication on ice. The homogenate was centrifuged at 14,000 g for 30 min at 4 °C to remove the remaining debris. Subsequently, four volumes of ice-cold acetone were added, and the proteins were precipitated at − 20 °C overnight. After centrifugation at 14,000 g for 30 min at 4 °C, the precipitate was collected and washed with ice-cold acetone three times. The collected protein pellets were dried with N_2_ to remove any remaining acetone. The dried protein powder was completely resuspended in 1 ml of dissolution buffer (8 M urea, 100 mM TEAB, pH 8.0). The resulting supernatant was reduced by adding 10 mM DTT. The reduction continued for 30 min at 56 °C. The sample was then alkylated with 55 mM iodoacetamide for 30 min at room temperature in darkness. The protein concentration was determined using the Bradford method [77].

### Trypsin digestion and iTRAQ labeling

For trypsin digestion, 100 μg of protein was diluted with four volumes of digestion buffer (100 mM TEAB, pH 8.0) and digested with 2 μg of Trypsin Gold (Promega, Madison, WI, USA) at 37 °C overnight. The digested peptides were desalted using a Strata-X C18 SPE column (Sigma, Shanghai, China), dried in a centrifugal vacuum concentrator and dissolved in 500 mM TEAB (pH 8.0) to allow for subsequent iTRAQ labeling. In this study, six peptide samples from shoots and stolons (each with three biological replicates) were labeled with iTRAQ tags following the manufacturer’s protocol for the iTRAQ® Reagents 8-PLEX Multiplex Kit (SCIEX, Framingham, MA, USA). Specifically, the three stolon samples were labeled with iTRAQ8–113, iTRAQ8–114 and iTRAQ8–115, whereas the three shoot samples were labeled with iTRAQ8–116, iTRAQ8–117 and iTRAQ8–118, respectively. The labeled peptides were pooled together, desalted using a Strata-X C18 SPE column, dried by vacuum centrifugation and fractionated using an Ultimate 3000 HPLC system (Dionex, Sunnyvale, CA, USA) with a Durashell C18 column (250 mm × 4.6 mm, 5 μm, 100 Å). Briefly, peptides were first fractionated with a gradient of 2–60% acetonitrile in ammonium bicarbonate (10 mM, pH 10) over 60 min into 60 fractions, which were further combined into 12 final fractions, desalted and vacuum dried.

### Liquid chromatography-tandem mass spectrometry (LC-MS/MS) analysis

For LC-MS/MS analyses, the fractionated peptides were dissolved in solvent A (0.1% formic acid in 2% acetonitrile, 98% H_2_O), loaded onto a reversed-phase precolumn (20 mm × 100 μm, 5 μm) and separated using a reversed-phase analytical column (150 mm × 75 μm, 3 μm) in an Eksigent nanoLC system (Eksigent, Livermore, CA, USA). The gradient for MS analysis was set as follows: starting at 7 to 20% solvent B (0.1% formic acid in 98% acetonitrile, 2% H_2_O) over 24 min, then 20–35% solvent B over 8 min, thereafter increasing to 80% solvent B over 55 min and holding at 80% solvent B for the last 3 min, with a flow rate of 300 nL/min. MS data were collected using information-dependent acquisition mode in a high speed TripleTOF™ 5600plus mass spectrometer (ABSciex, Concord, Canada) coupled with the Eksigent nanoLC system. The mass spectrometer was operated in a manner in which a 0.25 s survey scan (MS) in the mass range of 350–1500 m/z was collected, from which the top 30 ions were selected for automated MS/MS in the mass range of 100–1500 m/z and each MS/MS event consisted of a 0.05 s scan. Once a target ion had been fragmented by MS/MS, its mass and isotopes were excluded for a period of 15 s.

### Protein species identification and quantification

Analyses of the raw MS spectra generated by LC-MS/MS were performed with ProteinPilot™ 4.5 software (ABSciex) using the Paragon algorithm [78]. Specifically, a high-confidence peptide database (210,172 sequences, 69,486,195 residues) derived from a full-length transcriptome sequencing project of bermudagrass was used as the search database [79]. The searches were performed using the following settings: type of search, iTRAQ 8-PLEX (peptide labeled); enzyme, trypsin; Cys alkylation, iodoacetamide; instrument, TripleTOF™ 5600plus; bias correction, true; background correction, true; ID focus, biological modifications; search effort, thorough ID; protein mass, unrestricted; unused score, ≥ 1.3; confidence, ≥ 95%; unique peptides, ≥ 1. For protein species quantification, the quantitative protein ratios between shoots and stolons were weighted and normalized by the median ratio of the three replicates. Only ratios ≥1.5 or ≤ 0.67 and *p*-values≤0.05 in one-way ANOVA and Student-Newman-Keuls tests were considered as DAPs.

### Bioinformatics analyses of DAPs

The amino acid sequences of all the identified protein species were subjected to BLAST searches using the AmiGO database (http://amigo1.geneontology.org/cgi-bin/amigo/blast.cgi) and the KEGG database (http://www.kegg.jp/blastkoala/), respectively [80, 81]. The corresponding GO and KEGG terms were extracted from the most homologous proteins using a Perl program. The DAPs were searched against all the identified protein species using Fisher’s exact test in KOBAS with parameters set at an FDR-corrected *p* < 0.05 to obtain the significantly regulated biochemical pathways [82]. The amino acid sequences of the DAPs were also subjected to BLAST searches against the nucleotide sequence collections of differentially expressed unigenes previously reported with an E-value = 0 to identify the corresponding unigenes [25].

### RT-qPCR validation of DAPs

The frozen shoot and stolon internode samples described above were used for RNA extraction with an RNAprep Pure Plant Kit (Tiangen, Beijing, China). The RT-qPCR assays were performed using SYBR Premix ExTaq (TaKaRa, Dalian, China) and a Mini Opticon Real-Time PCR System (Bio-Rad, Hercules, USA). The actin gene (GenBank accession no. ES295104) was used as the standard control [83]. The primers used are listed in Additional file 3: Table S3. Each reaction was repeated three times. The relative gene expression level was calculated using the 2^–ΔΔCt^ method. Student’s t-test was used to determine the significance of the differences in gene expression levels.

### Western blotting

Antibodies against AGPase and PK were prepared by immunizing rabbits with synthesized protein-specific peptides (AGPase, PIPAAFCATGDLR; PK, LPKTKLVCTLGPA) mixed with Freund adjuvant by Beijing Protein Innovation (Beijing, China). Goat anti-rabbit antibodies conjugated to horseradish peroxidase were used as secondary antibodies (Abcam, ab97051). For western blotting, 20 μg protein samples prepared for LC-MS/MS analyses were denatured in 6 × SDS/PAGE sampling buffer by boiling at 100 °C for 5 min, then separated via 12% SDS/PAGE and transferred to a PVDF membrane. The signals were developed with a Lumi-light western blotting substrate (Roche, Mannheim, Germany). The experiments were repeated three times using different stolon and shoot protein samples.

### Enzyme activity assays

The shoot and stolon internodes were homogenized using a mortar and pestle with 100 mM MOPS-KOH buffer (pH 7.2) containing 2 mM EDTA, 5 mM DTT and 0.1% (w/v) PMSF. The homogenate was squeezed through three layers of cheesecloth and then centrifuged at 12,000 g for 15 min. The obtained soluble fraction was used for the enzyme activity assay. For the AGPase assay, the assay conditions were 100 mM HEPES-KOH (pH 7.9), 5 mM MgCl_2_, 0.4 mM NAD, 2.5 mM ADP-glucose, 1.5 mM Na_4_P_2_O_7_, 1 mM 3-phosphoglycerate, 10 units glucose-6-phosphate dehydrogenase (Sigma, Shanghai, China) and 4 units phosphoglucomutase (Sigma), in a final volume of 1 mL [26]. For the PK assay, the assay conditions were 50 mM HEPES–KOH (pH 6.4), 25 mM KCl, 12 mM MgCl_2_, 2 mM PEP, 1 mM ADP, 1 mM DTT, 5% (w/v) PEG8000, 0.15 mM NADH and 2 units lactate dehydrogenase (Sigma) in a final volume of 1 mL [27]. All reactions were assayed at 25 °C by monitoring the reduction of NAD or oxidation of NADH at 340 nm using an Ultrospec 3300 Pro spectrophotometer (Amersham Biosciences) with a continuous recording function. *V*_*max*_ and *K*_*m*_ values were automatically calculated by the spectrophotometer. The protein concentration was determined using the Bradford method. Tukey’s multiple comparison test was used to determine significant enzyme activity differences among the different plants.

## Supplementary information

**Additional file 1: Table S1.** Information on the 376 differentially accumulated protein species. (XLSX 49 kb)

**Additional file 2: Table S2.** Origin of the *Cynodon dactylon* cultivars and wild accessions. (XLSX 9 kb)

**Additional file 3: Table S3.** Primers used for RT-qPCR. (XLSX 9 kb)

**Additional file 4: Figure S1.** GO classification of the 376 differentially accumulated protein species. (TIF 2111 kb)

## Data Availability

The mass spectrometry data have been deposited to the ProteomeXchange Consortium via the PRIDE partner repository with the dataset identifier PXD012336.

## Abbreviations

2-DE: Two-dimensional gel electrophoresis
AGPase: ADP-glucose pyrophosphorylase
ALDO: Fructose-bisphosphate aldolase
C.V.: Coefficient of variation
CS: Cellulose synthase
DAP: Differentially accumulated protein
DEG: Differentially expressed unigene
FK: Fructokinase
GAPDH: Glyceraldehyde-3-phosphate dehydrogenase
GBE: 1,4-alpha-glucan-branching enzyme
GO: Gene ontology
ISA: Isoamylase
iTRAQ: isobaric tags for relative and absolute quantitation
KEGG: Kyoto Encyclopedia of Genes and Genomes
KOBAS: KEGG Orthology Based Annotation System
PAL: Phenylalanine ammonia-lyase
PFP: Pyrophosphate--fructose 6-phosphate 1-phosphotransferase
PGAM: Phosphoglycerate mutase
PK: Pyruvate kinase
PPDK: Pyruvate phosphate dikinase
SS: Starch synthase
SuS: Sucrose synthase
UGPase: UDP-glucose pyrophosphorylase
UV: Ultraviolet
