# Supplementary figures and images for: Comparative proteomic analysis provides new insights into the specialization of shoots and stolons in bermudagrass (Cynodon dactylon L.)
Source: BMC Genomics. 2019 Sep 11;20:708. doi: 10.1186/s12864-019-6077-3 (PMC6740039; doi:10.1186/s12864-019-6077-3)

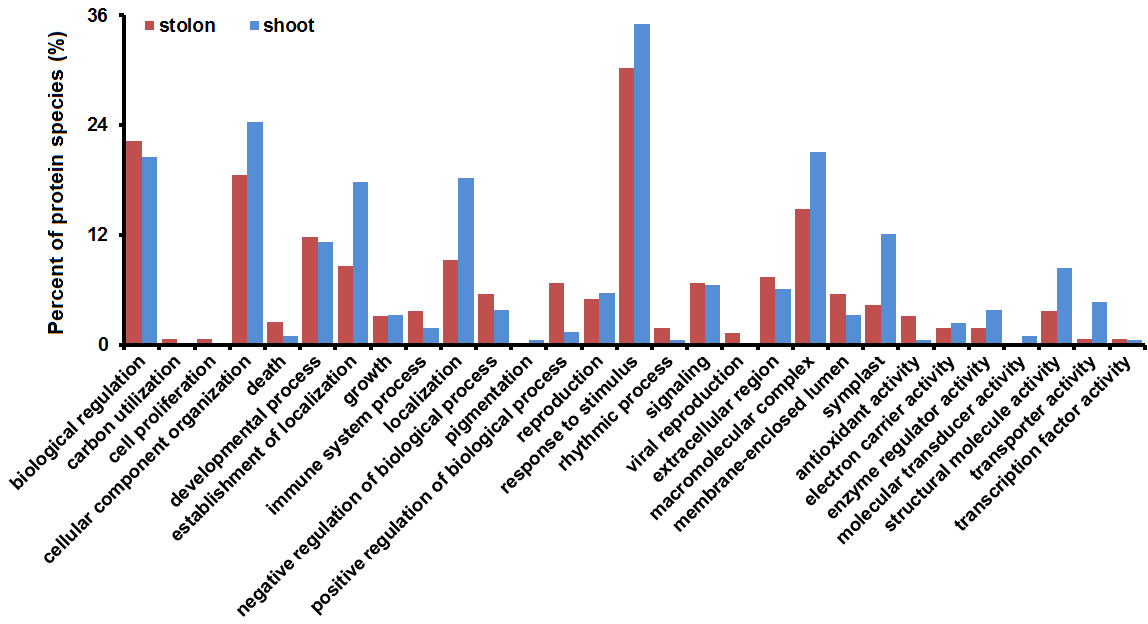

Supplement: Supplementary file 4 — Additional file 4: Figure S1. GO classification of the 376 differentially accumulated protein species. (TIF 2111 kb) [file 12864_2019_6077_MOESM4_ESM.tif]
